# Patterns of Comorbidity in Asthma–COPD Overlap Cohort According to Various ACO Definitions

**DOI:** 10.3390/arm94050062

**Published:** 2026-08-31

**Authors:** Andrzej Obojski, Krzysztof Kuziemski, Marek Barczyk, Rafał Krenke, Ewa Wycinka, Adam Barczyk

**Affiliations:** 1 Department of Allergology and Internal Medicine, Faculty of Medicine, Wroclaw Medical University, 50-556 Wroclaw, Poland; andrzej.obojski@poczta.onet.eu; 2 Department of Pulmonology, Collegium Medicum, University of Warmia and Mazury, 10-357 Olsztyn, Poland; 3 Department of Computer Networks and Systems, Silesian University of Technology, 44-100 Gliwice, Poland; barczyk@gmail.com; 4 Department of Internal Medicine, Pulmonary Diseases & Allergy, Medical University of Warsaw, 02-097 Warsaw, Poland; rafalkrenke@interia.pl; 5 Department of Statistics, Faculty of Management, University of Gdansk, 81-824 Sopot, Poland; ewa.wycinka@ug.edu.pl; 6 Department of Pneumonology, Faculty of Medical Sciences in Katowice, Medical University of Silesia, 40-635 Katowice, Poland; adagne@icloud.com

**Keywords:** asthma–COPD overlap definition, ACO, comorbidity

## Abstract

**Highlights:**

**What are the main findings?**

- Chronic sinusitis is a comorbidity that consistently occurs more frequently in ACO groups defined by more precise criteria of asthma, i.e., GINA/GOLD/30% group, Spanish criteria group, and COPD + asthma < 40 yrs group.
- This may be a consequence of the causal relationship between rhinosinusitis and asthma.

**What are the implications of the main findings?**

- The definitions of ACO used in clinical practice vary widely and describe different populations.
- ACO populations vary in terms of comorbidities, lung function, smoking history, and age.

**Abstract:**

**Background:** The asthma–COPD overlap is widely recognized, but its characteristics are not fully understood. This is largely true for the clinical presentation characterized by various ACO definitions. The purpose of this study was to investigate the pattern of comorbidities among patients with ACO, defined by various criteria. **Methods:** The data came from the multicenter cross-sectional study conducted in a mixed population of patients with asthma and COPD. Patients were classified into five groups according to five different definitions of ACO (GINA/GOLD/30%, Spanish criteria, COPD + asthma < 40, Gibson’s criteria, clinician’s diagnosis), reference ACO groups, and a group of patients with obstructive pulmonary diseases who were not diagnosed with ACO. To demonstrate the differences between groups, we compared the prevalence of comorbidities between all ACO and non-ACO groups. Data on age, severity of airflow obstruction, and smoking history were recorded for all patients. **Results:** For the final analysis, 1609 patients were included. Patients belonging to the ACO groups had a higher burden of comorbidities, poorer lung function, and a higher number of pack-years of smoking history compared to patients without an ACO diagnosis. Comorbidities were significantly more prevalent in ACO groups. Compared to the non-ACO group, nine diseases were more common in the GINA/GOLD/30% group, seven in both the COPD + asthma < 40 group and the clinician’s diagnosis group, five in the Gibson’s criteria group, and two in the Spanish criteria group. The most common comorbidities in the ACO groups were hypertension, coronary artery disease, GERD, musculoskeletal disorders, diabetes, and chronic sinusitis. Chronic sinusitis was more frequent in ACO groups defined by more precise asthma criteria. **Conclusions:** The definitions of ACO used in clinical practice vary widely and describe different populations. ACO populations vary in terms of comorbidities, lung function, smoking history, and age. Chronic sinusitis is a comorbidity that occurs more frequently in ACO groups defined by more precise asthma criteria. Patients with ACO differ in their clinical outcomes from those with other obstructive airway diseases.

## 1. Introduction

Asthma and chronic obstructive pulmonary disease (COPD) are the two most common obstructive airway diseases. Despite many pathophysiological similarities, they are well-defined as two distinct disorders [1,2]. Asthma and COPD share numerous clinical features, including bronchial hyperresponsiveness, airway obstruction, and symptoms. The overlap and convergence in the clinical presentation can complicate differential diagnosis, particularly in elderly patients with late-onset disease and irreversible airway obstruction. It has also been acknowledged that some patients exhibit clinical phenotypes combining features of both asthma and COPD, a condition termed the asthma–COPD overlap syndrome (ACO) [3]. Although the coexistence of COPD and asthma is widely recognized, it remains poorly characterized. There is insufficient knowledge regarding the prevalence of ACO, its associated risk of hospitalization, as well as patterns of comorbidities. Assessing the prevalence of ACO is challenging, primarily due to the lack of standardized diagnostic criteria. It is estimated that ACO accounts for 15–25% of patients with obstructive airway disease [4]. Exacerbations of COPD contribute significantly to morbidity, mortality, and the economic burden [5]. It is recognized that individuals with ACO have a higher risk of exacerbations than those with COPD alone [6,7]. The number of emergency room (ER) visits, hospital admissions, and intensive care unit (ICU) admissions is significantly higher in ACO than in COPD, which corresponds to increased healthcare utilization and cost [8]. The higher rates of exacerbations, hospitalizations, and medical expenses indicate a more severe course of ACO compared to COPD. The role and significance of comorbidities in ACO is still under clinical investigation. Even less is known about the characteristics and clinical presentation of patients with ACO defined by various definitions of ACO.

The purpose of this study was to examine the patterns of comorbidities in patients with ACO, as defined by different diagnostic criteria. We hypothesized that patient groups identified based on varying definitions of ACO would differ in terms of their comorbidity profiles, revealing distinct characteristics within the study populations.

## 2. Materials and Methods

The data used in this analysis were derived from a multicenter cross-sectional study. The data were collected by pulmonary specialists using a questionnaire designed solely for the study. The target population comprised patients over 35 years diagnosed with asthma, COPD, chronic bronchitis, or emphysema. The final dataset included 1609 patients, 808 women and 801 men, with a mean age of 62.6 ± 11.6 years. Findings on the concordance among different ACO definitions, as well as the prevalence and characteristics of ACO populations, were presented in a previous publication [9]. The study also collected data on the prevalence of comorbidities, which have not yet been published.

Five commonly recognized ACO definitions were used: 1/ GINA/GOLD criteria [3], 2/ the Spanish criteria proposed by Soler-Cataluna et al. [10], 3/ current diagnosis of COPD in a patient with asthma diagnosed before the age of 40 years [7,11,12], 4/ the definition proposed by Gibson and Simpson [13], and 5/ physician’s diagnosis based on own clinical experience and judgment. The key diagnostic criteria comprising the various ACO definitions used in the present study are summarized in Table 1.

**Figure 1:**
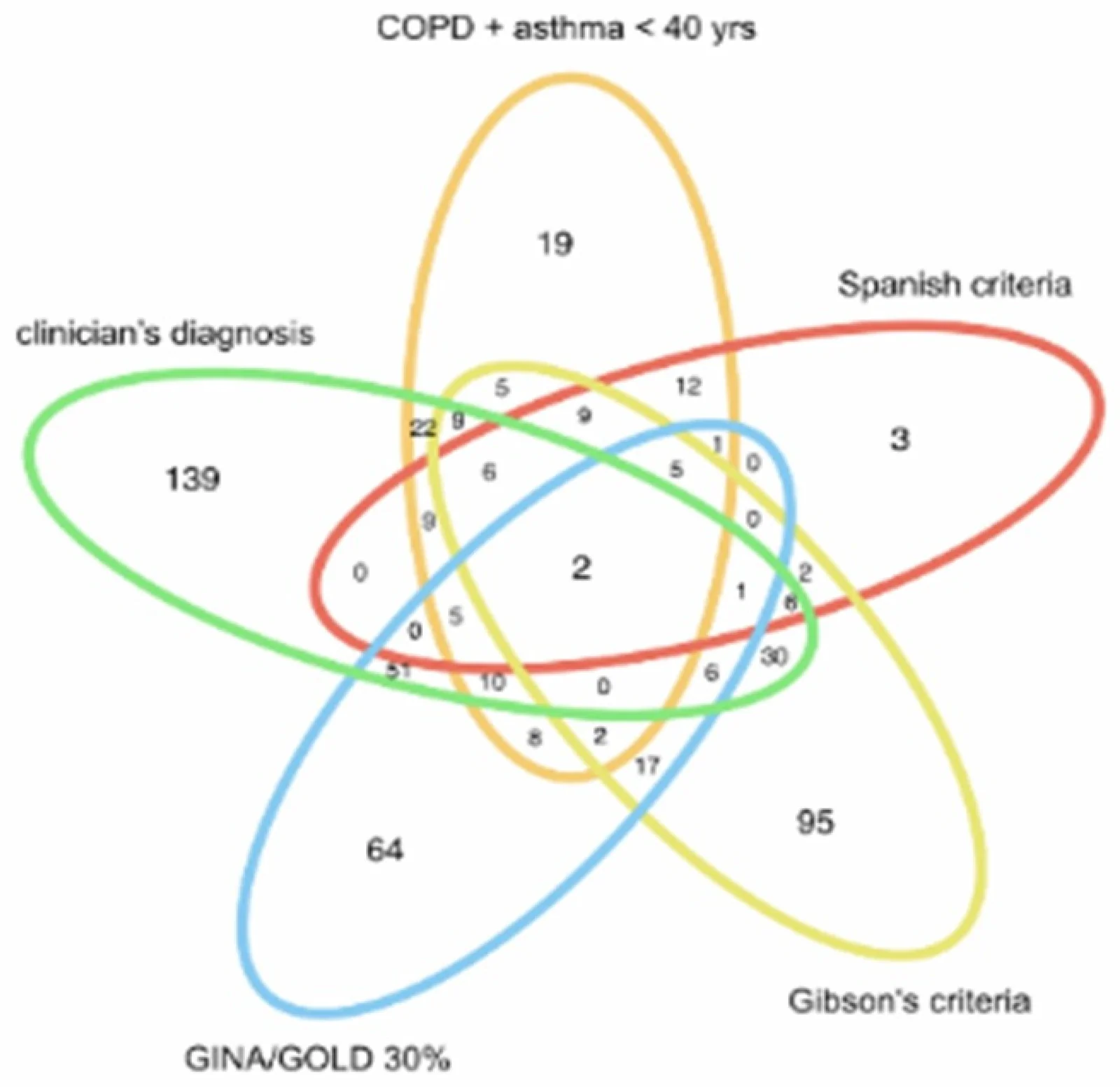
The elliptical diagram of the ACO groups and group overlap according to various definitions [9].

**Figure 2:**
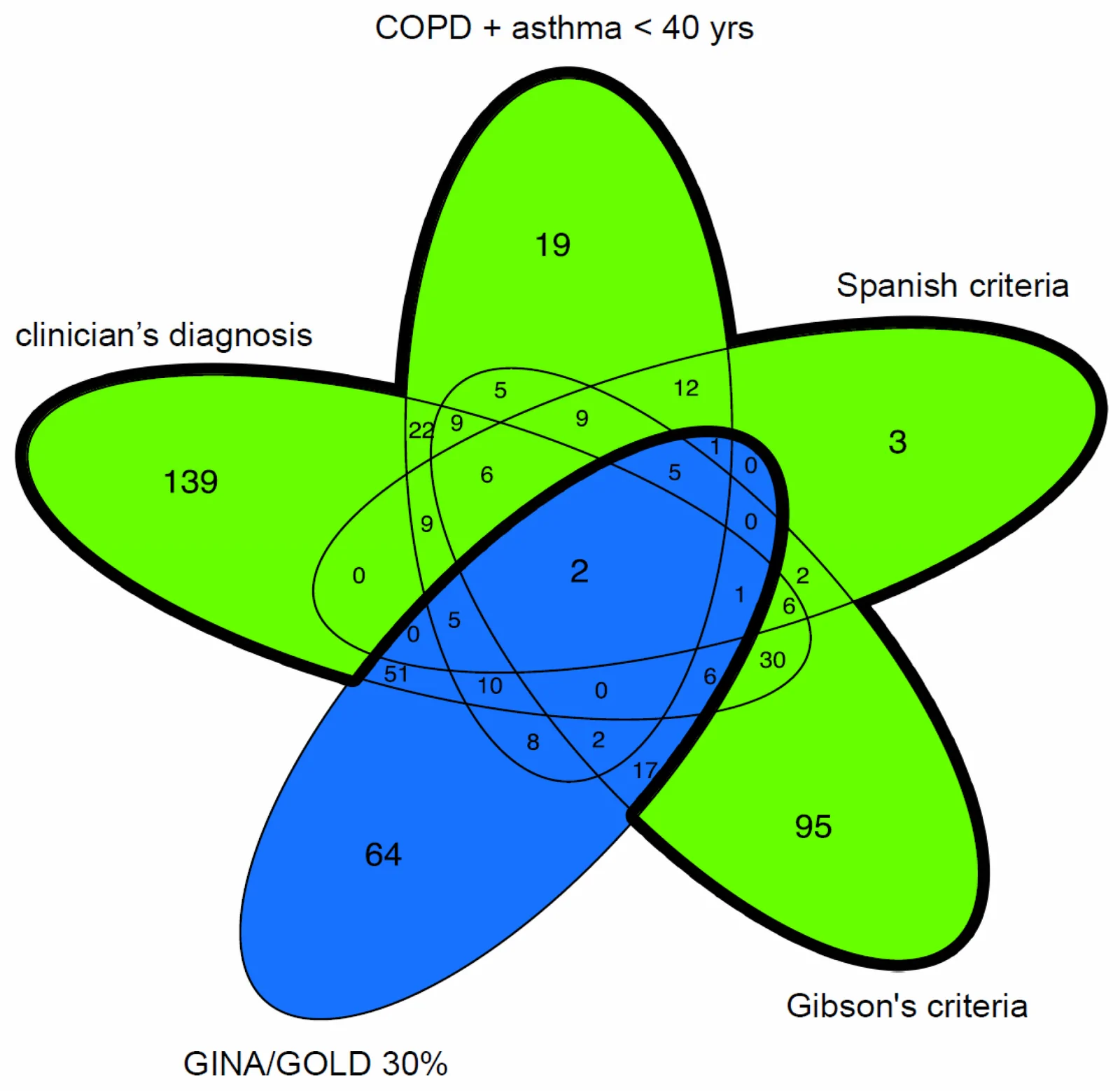
Diagram illustrating the creation of the reference “other-ACO” group (green) using the GINA/GOLD/30% group (blue) as an example.

The patient groups identified by each definition were labeled as GINA/GOLD/30% group, Spanish criteria group, COPD + asthma < 40 yrs group, Gibson’s criteria group, and clinician’s diagnosis group, respectively. Details concerning the basic study design, population, and collected data are provided in Barczyk et al. [9]. For the purposes of this study, additional reference groups were created. For each of the five ACO groups, a corresponding “other-ACO” reference group was formed, comprising patients who met at least one of the remaining four ACO definitions but did not meet the criteria of the index group. For example, the “other-ACO” group for the GINA/GOLD/30% group included patients fulfilling any of the Spanish criteria, COPD + asthma < 40 yrs criteria, Gibson’s criteria, and/or clinician’s diagnosis, but not meeting the GINA/GOLD/30% criteria. An example of the relationship between the ACO group and its respective “other-ACO” reference group is illustrated for the GINA/GOLD/30% group in Figure 1 and Figure 2. This approach enabled comparison of comorbidity prevalence across ACO populations defined by different criteria.

A new non-ACO group was also created, comprising all patients who did not meet any of the five ACO definitions. This group included individuals diagnosed with asthma, COPD, chronic bronchitis, emphysema, and other chronic respiratory diseases. The combination of all chronic obstructive respiratory disorders created a unique group exhibiting features of asthma and COPD, which to some extent mimics the characteristics of ACO. In addition, the combination of different obstructive pulmonary diseases allowed us to minimize the impact of age and smoking history on the prevalence of comorbidities within this group. Based on these criteria, we compared the prevalence of comorbidities between each ACO group and the non-ACO group. Finally, we compared the prevalence of comorbidities between the all-ACO group, i.e., all patients who met at least one ACO criterion, and the non-ACO group, i.e., all patients without the diagnosis of ACO. We hypothesized that this approach could reliably demonstrate the potential differences in the prevalence of comorbidities both across ACO-defined subpopulations and between the overall ACO population and the broader group of patients with other obstructive respiratory diseases.

Basic data on age, the severity of airway obstruction (measured as a post-bronchodilator forced expiratory volume in one second, expressed as the percent of the predicted value; FEV_1_%pred), and smoking history (measured in pack-years; PY) were recorded for all patients. GLI-2012 reference values were used to interpret spirometry test results [14]. Comorbidities included in the final dataset are presented in Table 2.

### Statistical Analysis

Statistical analysis was conducted using the methods described in the previous study [9]. All analyses were performed using the R language and environment for statistical computing. The normality of the distributions of continuous variables was assessed by the Shapiro–Wilk test and using q-q plots.

The planned comparison framework included: (i) each ACO group versus its corresponding other-ACO reference group, (ii) each ACO group versus the non-ACO group, and (iii) the all-ACO group versus the non-ACO group. For each comparison, the prevalence of all 33 recorded comorbidities was evaluated.

Categorical variables were presented as percentages within groups, whereas continuous variables were presented as means and SD. Statistical significance of differences between categorical variables was analyzed using the chi-square test, followed by Fisher’s exact test for comparisons of 2 groups. The differences among groups in terms of continuous variables were analyzed using the Kruskal–Wallis rank sum test, followed by the Mann–Whitney U test for multiple group comparisons.

The present study was designed as an exploratory analysis intended to characterize patterns of comorbidities across patient populations identified by different ACO definitions rather than to test a limited number of prespecified hypotheses. Therefore, all reported *p*-values should be considered nominal *p*-values. No formal adjustment for multiple comparisons was applied. This approach was adopted to avoid excessive inflation of type II error and potential masking of clinically relevant associations in an exploratory setting, and is consistent with the recommendations of Rothman as well as subsequent methodological discussions regarding exploratory medical research [15,16]. Importantly, to minimize the risk of selective reporting and overestimation of false-positive findings, all analyzed outcomes are reported, including statistically non-significant results. Thus, the presented data reflect the complete set of performed analyses rather than a selected subset of statistically significant findings. A *p*-value of less than 0.05 was considered statistically significant.

## 3. Results

Patients with ACO, as defined by the five ACO definitions, differed from those with other obstructive respiratory diseases (non-ACO group) in clinical characteristics. The Spanish criteria group was younger than the non-ACO group. In contrast, patients in Gibson’s criteria and the clinician’s diagnosis group were older than those in the non-ACO group. The age of patients in the GINA/GOLD/30% group and COPD + asthma < 40 yrs group did not differ significantly from the non-ACO group. There were also differences in the severity of the airflow limitation between groups (Table 3). The mildest obstruction was observed in the non-ACO group with a mean post-BD FEV_1_%pred of 68.6 ± 24.6%. All ACO groups had significantly lower mean post-BD FEV1%pred values than the non-ACO group. They were as follows (*p*-value vs. non-ACO group):

- The GINA/GOLD/30% group 64.1 ± 19.0% (*p* = 0.033),
- The Spanish criteria group 60.5 ± 17.6% (*p* = 0.009),
- The COPD + asthma < 40 yrs group 56.9 ± 17.3% (*p* < 0.001),
- The clinician’s diagnosis group 58.5 ± 19.1% (*p* < 0.001),
- The Gibson’s criteria group 53.8 ± 16.3% (*p* < 0.001).

Interestingly, an opposite pattern of data distribution of lung function was observed for smoking history. The lowest cumulative tobacco exposure was recorded in the non-ACO group, with a mean of 16.9 ± 22.7 PY. In all ACO groups, the number of PY was significantly higher than in the non-ACO group:

- Clinician’s diagnosis group 20.2 ± 21.9 PY (*p* = 0.001),
- GINA/GOLD/30% group 19.3 ± 22.8 PY (*p* = 0.014),
- Spanish criteria group 22.4 ± 19.3 PY (*p* < 0.001),
- COPD + asthma < 40 yrs group 23.3 ± 18.3 PY (*p* < 0.001),
- Gibson’s criteria group 30.7 ± 22.1 PY (*p* < 0.001).

The data on age, severity of airflow limitation, and smoking history are summarized in Table 3.

Comparison of comorbidity prevalence between ACO groups and their corresponding reference “other-ACO” groups showed several significant differences. In the GINA/GOLD/30% group, a higher prevalence was found for chronic sinusitis (16.9% vs. 8.5%, *p* = 0.005), other than GERD gastrointestinal tract diseases (14.5% vs. 7.9%, *p* = 0.021), endocrine diseases (13.4% vs. 6.6%, *p* = 0.013), and anemia (2.9% vs. 0.6%, *p* = 0.037). Conversely, this group demonstrated a lower prevalence of myocardial infarction (4.1 vs. 9.6, *p* = 0.026) and tuberculosis (1.2 vs. 5.5, *p* = 0.018). In the Spanish criteria group, GERD (34.4% vs. 21.2%, *p* = 0.024) and chronic sinusitis (19.7% vs. 10.1%, *p* = 0.031) were significantly more common than in the reference “other-ACO” group. In the COPD + asthma < 40 yrs group, Gibson’s criteria group, and clinician’s diagnosis group, there was only one disease with a higher prevalence compared to their corresponding “other-ACO” groups. These were chronic sinusitis (17.7% vs. 9.2%, *p* = 0.014), tuberculosis (6.7% vs. 2.6%, *p* = 0.039), and polycythemia (3.0% vs. 0%, *p* = 0.005), respectively.

**Figure 3:**
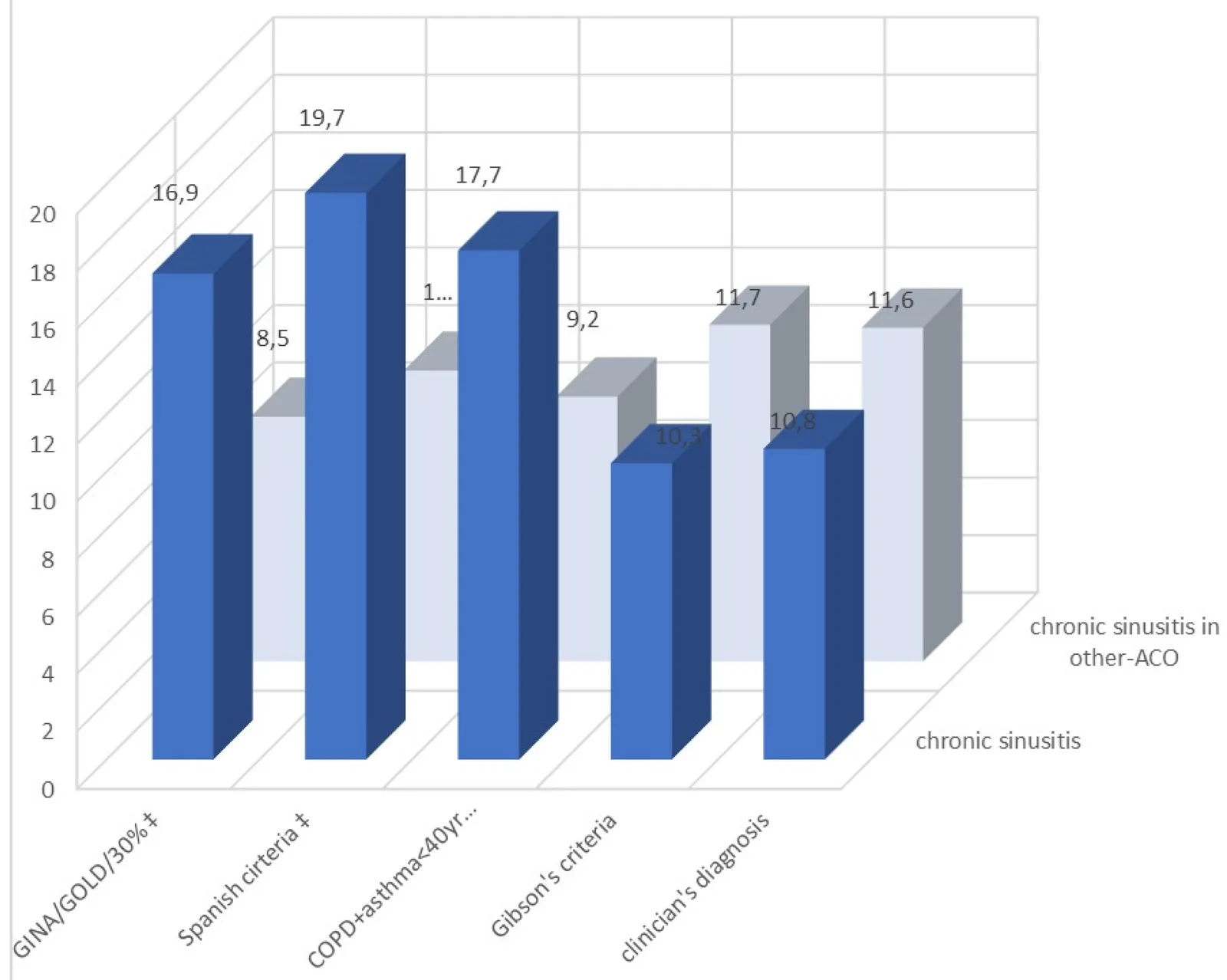
The prevalence of chronic sinusitis in the GINA/GOLD/30% group, the Spanish criteria group, the COPD + asthma < 40 yrs group, the Gibson’s criteria group, and the clinician’s diagnosis group, and respective reference “other-ACO” groups. ‡ statistically significant.

Additionally, in Gibson’s group, the prevalence of GERD was lower compared to the reference “other-ACO” group (17.4 vs. 25.7, *p* = 0.032). It is worth noting that chronic sinusitis occurred consistently more frequently in some ACO groups. This applies to the GINA/GOLD/30% group, the Spanish criteria group, and the COPD + asthma < 40 yrs group. The differences in the prevalence of chronic sinusitis between the ACO groups and the reference “other-ACO” groups are presented in Figure 3. Comprehensive data on the prevalence of all comorbidities included in the dataset in the ACO groups and the reference “other-ACO” groups are presented in the online Supplementary Table S1.

We also compared the prevalence of comorbidities between the ACO groups and the non-ACO group. Comorbidities were consistently more common in all ACO groups. Compared to the non-ACO group, nine diseases were more common in the GINA/GOLD/30% group, seven diseases in the COPD + asthma < 40 yrs group and the clinician’s diagnosis group, five diseases in the Gibson’s criteria group, and two diseases in the Spanish criteria group. The detailed differences in the prevalence of comorbidities between the ACO and non-ACO groups (*p*-value) are presented below.

In the GINA/GOLD/30% group: hypertension (65.7% vs. 50.1%, *p* < 0.001), GERD (27.9 vs. 16.4, *p* = 0.001), musculoskeletal disorders (18.6% vs. 9.6%, *p* < 0.001), chronic sinusitis (16.9% vs. 8.3%, *p* = 0.001), other than GERD gastrointestinal disease (14.5% vs. 7.9%, *p* = 0.009), genitourinary diseases (12.8% vs. 6.1%, *p* = 0.003), osteoporosis (11.1% vs. 5.0%, *p* = 0.004), insomnia (9.9% vs. 3.6%, *p* = 0.001) and ophthalmic diseases (7.6% vs. 3.2%, *p* = 0.009).

In the COPD + asthma < 40 yrs group: hypertension (62.8% vs. 50.1%, *p* = 0.022), GERD (25.0 vs. 16.4, *p* = 0.023), chronic sinusitis (17.7% vs. 8.3%, *p* = 0.002), osteoporosis (12.1% vs. 5.0%, *p* = 0.003), myocardial infarction (11.3% vs. 5.3%, *p* = 0.014), depression (8.9% vs. 4.2%, *p* = 0.039) and ophthalmic diseases (8.1% vs. 3.2%, *p* = 0.019).

In the clinician’s diagnosis group: hypertension (61.2% vs. 50.1%, *p* < 0.001), coronary artery disease (30.4% vs. 20.6%, *p* < 0.001), GERD (22.3 vs. 16.4, *p* = 0.025), musculoskeletal disorders (16.9% vs. 9.6%, *p* < 0.001), osteoporosis (10.5% vs. 5.0%, *p* < 0.001), insomnia (7,8% vs. 3.6%, *p* = 0.004) and polycythemia (3.0% vs. 0.8%, *p* = 0.004).

In the Gibson’s criteria group: hypertension (61.5% vs. 50.1%, *p* = 0.004), musculoskeletal disorders (14.9% vs. 9.6%, *p* = 0.031), genitourinary diseases (11.8% vs. 6.1%, *p* = 0.008), myocardial infarction (9.2% vs. 5.3%, *p* = 0.046), and tuberculosis (6.7% vs. 2.6%, *p* = 0.007).

In the Spanish criteria group: GERD (34.4% vs. 16.4%, *p* < 0.001) and chronic sinusitis (19.7% vs. 8.3%; *p* = 0.008).

**Figure 4:**
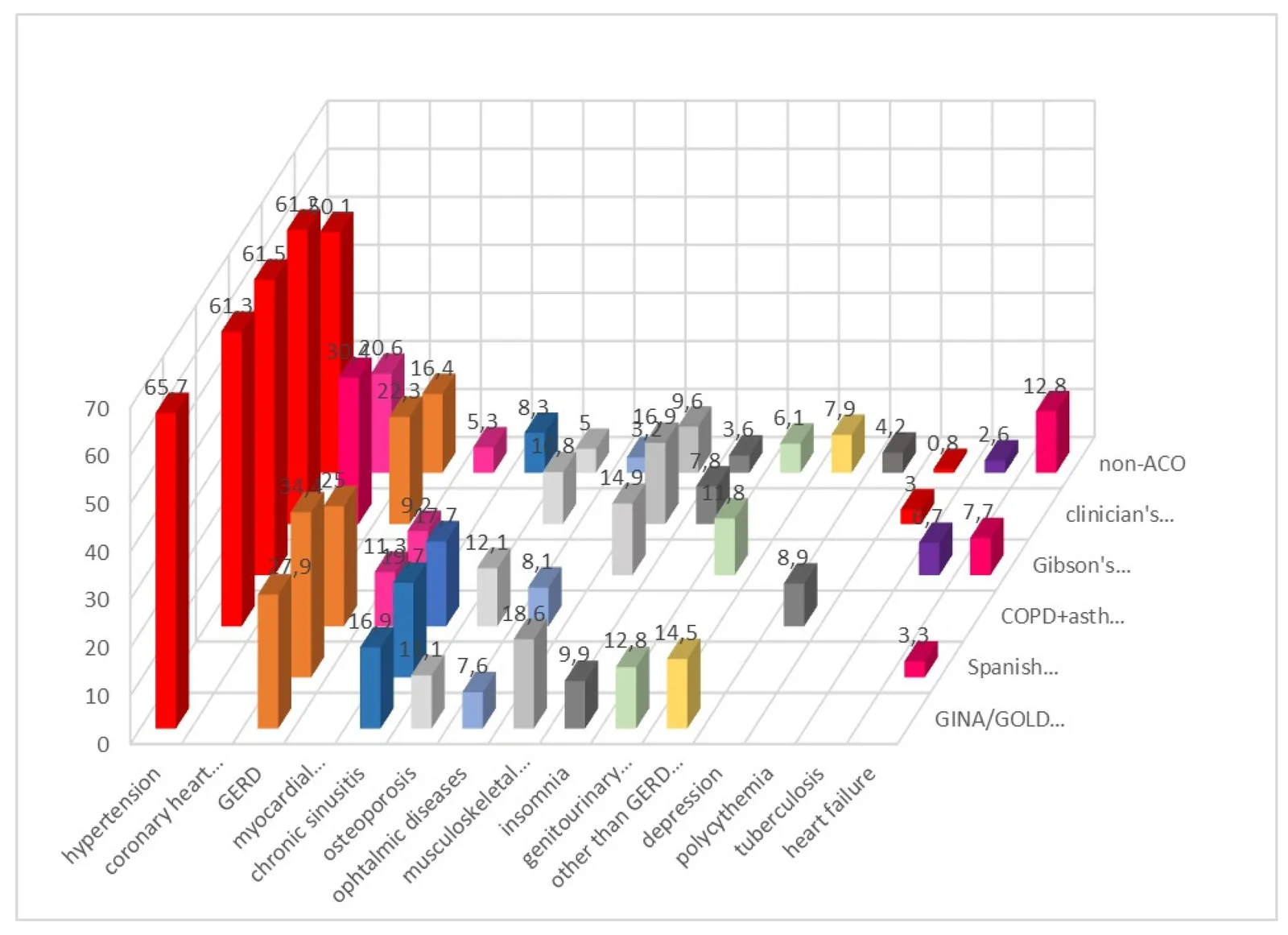
The prevalence of comorbidities in ACO groups and the non-ACO group. Disease prevalence is expressed as a percentage of patients in a group diagnosed with a particular disease [%]. All reported *p*-values are nominal *p*-values.

Heart failure was the only comorbidity found to be significantly more prevalent in the non-ACO group compared to the ACO groups: Non-ACO: 12.8%, Gibson’s criteria group: 7.7% (*p* = 0.042), Spanish criteria group: 3.3% (*p* = 0.026). The data are summarized in Figure 4. Complete details on the prevalence of all comorbidities across ACO groups and the non-ACO group are available in the online Supplementary Table S1.

**Figure 5:**
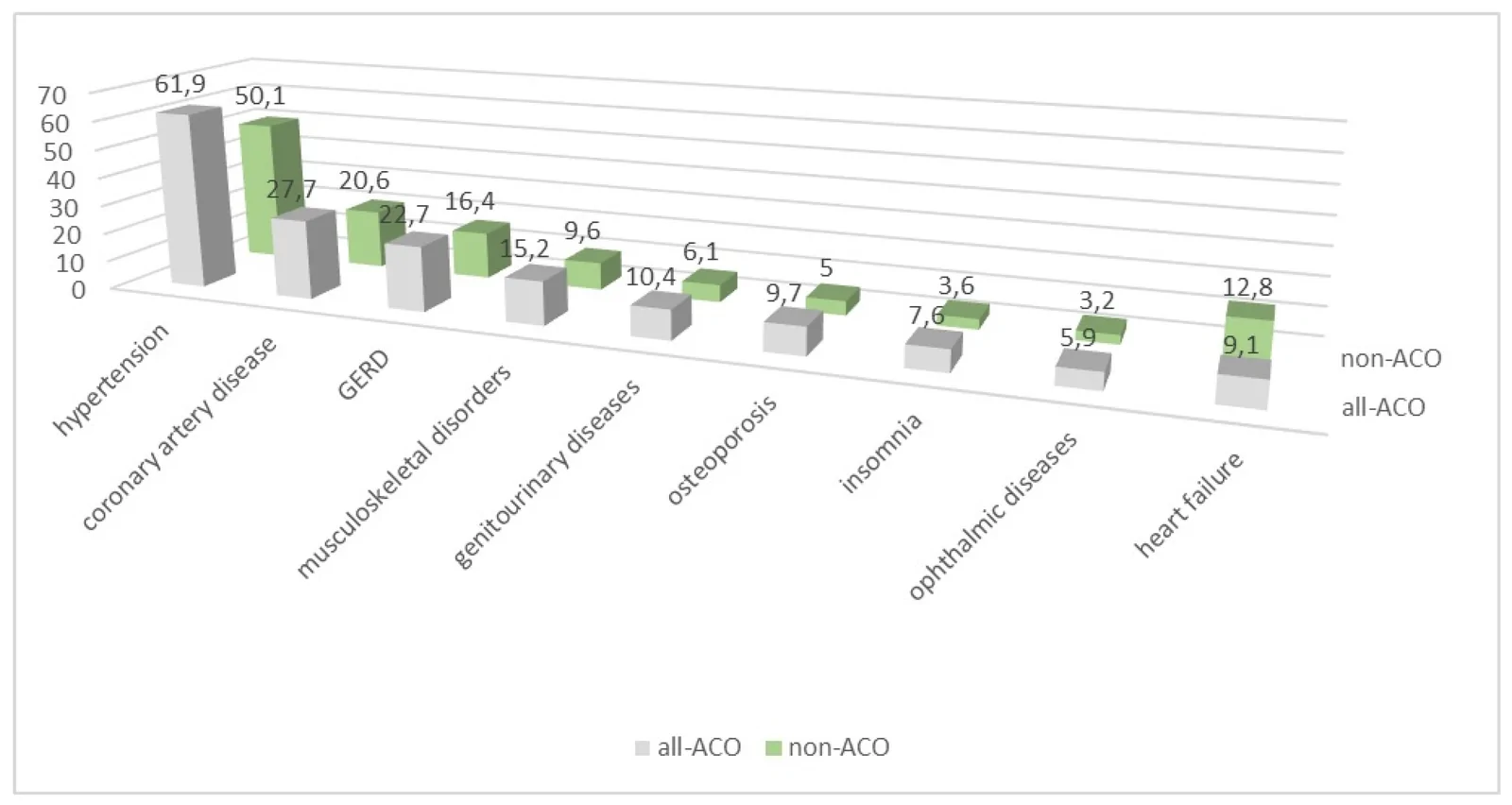
The prevalence of comorbidities in the all-ACO group and non-ACO group. Disease prevalence is expressed as a percentage of patients in a group diagnosed with a particular disease [%]. All reported *p*-values are nominal *p*-values.

In the final step of the analysis, we compared the prevalence of comorbidities between the all-ACO group (i.e., patients meeting at least one ACO definition) and the non-ACO group (i.e., patients meeting none of the ACO definitions). The prevalence of eight comorbidities was higher in the all-ACO group than in the non-ACO group: hypertension 61.9% vs. 50.1% (*p* < 0.001), coronary artery disease 27.7% vs. 20.6% (*p* = 0.002), gastroesophageal reflux disease 22.7% vs. 16.4% (*p* = 0.003), musculoskeletal disorders 15.2% vs. 9.6% (*p* = 0.002), genitourinary diseases 10.4% vs. 6.1% (*p* = 0.003), osteoporosis 9.7% vs. 5.0% (*p* < 0.001), insomnia 7.6% vs. 3.6% (*p* < 0.001) and ophthalmic diseases 5.9% vs. 3.2% (*p* = 0.011), respectively. Heart failure was the only comorbidity found to be significantly less prevalent in the all-ACO group than in the non-ACO group: 9.1% vs. 12.8%, respectively (*p* = 0.032). The results are illustrated in Figure 5. Complete data on the prevalence of all comorbidities in the all-ACO group and the non-ACO group are presented in the online Supplementary Table S2.

## 4. Discussion

Unless otherwise stated, references to statistical significance throughout the text refer to nominal *p*-values from exploratory analyses. A potential difference between the ACO groups may be the presence of distinct comorbidities. To verify this assumption, we compared each ACO group separately to the reference other-ACO group that included all other patients diagnosed with ACO. The other-ACO group serves as a reference group that encompasses merged features of ACO patients. To our knowledge, such analysis has not been the subject of research so far. The data analysis supported the adopted assumption. ACO groups differed from each other in the prevalence of comorbidities. Comorbidities with higher prevalence than in the other-ACO reference groups were more common in ACO groups that more precisely meet asthma criteria. In the GINA/GOLD/30% group, the diagnoses were chronic sinusitis, other than GERD, gastrointestinal tract diseases, endocrine diseases, and anemia, whereas in the Spanish criteria group, the diagnoses were GERD and chronic sinusitis. Gibson’s criteria group, clinician’s diagnosis group, and COPD + asthma < 40 group were associated with only one comorbidity that was more prevalent than in the other-ACO reference group. It was tuberculosis, polycythemia, and chronic sinusitis, respectively. Also noteworthy is the lower prevalence of tuberculosis and myocardial infarction in the GINA/GOLD/30% group and GERD in the Gibson’s group. The question of whether the comorbidity might be related to the nature of the ACO definition, which emphasizes features of COPD or asthma, remains unanswered. However, prior pulmonary tuberculosis is a risk factor for COPD [17], but not for asthma. There is also an association between polycythemia and advanced COPD associated with chronic respiratory failure, but not asthma. Polycythemia is one of the criteria for initiating long-term oxygen therapy in COPD [18]. Anemia, especially anemia of chronic disease, is another common comorbid disease in COPD [19,20,21] and not in asthma. Similarly, coronary heart disease and myocardial infarction are associated with COPD, not asthma [22,23]. Gastroesophageal reflux disease (GERD) is a contributory comorbidity in both asthma and COPD. GERD is a recognized risk factor for poor asthma control and exacerbations [24] and an independent risk factor for exacerbations of COPD [25]. Other than GERD, gastrointestinal tract diseases and endocrine diseases are less precisely defined entities, making the assessment of the potential association with obstructive diseases uncertain. In conclusion, it appears that the nature of the ACO definition, emphasizing features of COPD or asthma, may be related to differences in comorbidities.

Chronic sinusitis is a significant comorbidity in asthma–COPD overlap as it occurs consistently across most of ACO groups. Interestingly, this applies to the ACO groups based on definitions that more precisely describe the characteristics of asthma, i.e., GINA/GOLD/30% group, Spanish criteria group, and COPD + asthma < 40 group. The higher prevalence of chronic sinusitis observed in patients with ACO may be interpreted within the framework of the “united airway disease” concept, which emphasizes the close anatomical, physiological, and immunological relationship between the upper and lower airways. Chronic rhinosinusitis and asthma share common inflammatory pathways, including epithelial dysfunction, impaired mucociliary clearance, and activation of overlapping immune mechanisms. In particular, type 2 inflammatory processes, involving cytokines such as IL-4, IL-5, and IL-13, may contribute to the coexistence of upper and lower airway manifestations in a subset of patients with chronic airway diseases.

The presence of COPD-related features may further contribute to this complex inflammatory phenotype through additional mechanisms, including persistent airway inflammation, increased exposure to environmental risk factors, and structural airway changes. Therefore, the increased frequency of chronic sinusitis in patients with ACO may reflect the coexistence of shared inflammatory mechanisms and a greater overall burden of airway disease rather than representing an isolated comorbidity. These findings highlight the importance of assessing upper airway manifestations as part of the comprehensive clinical evaluation of patients with ACO. Chronic sinusitis was shown to be associated with the frequency of asthma exacerbations [24] and identified as an independent risk factor for asthma exacerbations [26]. Chronic sinusitis may contribute to poor asthma control and play a causal role in difficult-to-treat asthma [26]. Treatment of rhinitis and sinusitis with intranasal steroids was shown to reduce exacerbations of asthma, leading to emergency department visits [27]. Allergic sinusitis plays a significant role in asthma. Allergic rhinosinusitis, especially if uncontrolled, is associated with an increased risk of asthma and is a powerful predictor of adult-onset asthma [28]. A similar observation also applies to non-allergic rhinosinusitis [28]. This led to the development of the united airways concept, with similar types of inflammatory processes in the upper and lower respiratory tract [29,30]. In COPD, rhinitis and sinusitis are also common. In a large epidemiological study, it was shown that 40% of subjects with COPD had significant nasal symptoms, and nasal symptoms were more common among smokers than nonsmokers [31]. In another study, in a smaller group of COPD patients, chronic nasal symptoms occurred in up to 75% of patients [32]. Rhinitis and chronic sinusitis clearly coexist with COPD; however, evidence suggests that smoking alone rather than systemic inflammation triggers the condition [33]. The clinical relationship between chronic sinusitis and COPD is not fully understood. Associations were found between nasal symptoms and daily sputum production as well as between post-nasal drip and sputum production, but no significant relationship was found between nasal symptoms and any other lower respiratory tract symptom [32]. It was shown that the degree of nasal airway obstruction in COPD reflects impaired pulmonary airflow [34], but no association was found between nasal symptoms and lung function (FEV1) [31]. The treatment of chronic nasal symptoms with inhaled nasal budesonide may have a beneficial effect on COPD symptoms and quality of life but not on pre-bronchodilator spirometric parameters [35]. The link between chronic sinusitis and COPD appears to be symptomatic rather than causal and is due to exposure to common harmful agents.

The data presented confirm that the populations of patients with overlap of asthma and COPD, selected using different definitions of ACO, differ in comorbidities. Patients who meet ACO definitions that define asthma less precisely are more likely to suffer from comorbidities associated with COPD, such as tuberculosis, polycythemia, and anemia, while patients who meet ACO definitions that include basic criteria for asthma are less likely to have tuberculosis and myocardial infarction and more likely to suffer from chronic sinusitis, an asthma-related condition.

The above observations raise the question of the extent to which differences in ACO definitions lead to the emergence of distinct groups of patients with significantly different clinical parameters.

Our study was not designed to address this question; however, we noted that the pattern of comorbidity is associated with some of the other discriminative clinical characteristics of ACO groups. The ACO groups differ in terms of post-bronchodilator FEV1%pred, smoking pack-years, and age. The Gibson’s group presents the lowest post-bronchodilator FEV1%pred and the highest smoking pack-year history of all ACO groups. Conversely, the GINA/GOLD/30% group shows the highest post-bronchodilator FEV1%pred and the smallest number of pack-years. Spanish criteria group and the COPD + asthma < 40 yrs group, like the GINA/GOLD/30% group, present higher post-bronchodilator FEV1%pred and a smaller number of pack-years. A similar pattern can be found in terms of age. Patients in the Gibson’s group and clinicians’ diagnosis group were older, while in the Spanish criteria, COPD + asthma < 40 yrs, and GINA/GOLD/30% groups were younger. The distribution of clinical features, including lung function, smoking history, age, and comorbidities, aligns with the characteristics of COPD or asthma. The diverse populations of ACO patients may be a consequence of the discriminatory nature of the ACO definitions.

In the next stage of the study, we compared the ACO groups with the non-ACO group. Comparison of comorbidities between different ACO groups and the heterogeneous group of obstructive diseases is a novel approach. This original and previously undescribed analysis was proposed to better reveal the nature of ACO. Combining patients with asthma, COPD, and other obstructive diseases into one non-ACO group created a unique reference population sharing the features of asthma and COPD. Assuming that ACO is a simple co-occurrence of asthma and COPD, comparing the ACO groups and the non-ACO group should not result in a notable difference. The proposed analysis is a voice in the discussion on the essence of ACO. The term asthma–COPD overlap syndrome (ACOS) was introduced in 2009 by Gibson and Simpson [13] and has evolved significantly in recent years. In 2017, the syndrome was reduced to a simple ACO [36] to emphasize that it is not a separate nosological unit but a kind of comorbidity. Finally, in 2020, the term was entirely removed from the GOLD report to further emphasize that asthma and COPD are different entities and their co-occurrence does not bring a new significant quality [37]. Our data indicate that regardless of the definition of ACO, the asthma–COPD overlap differs significantly from the obstructive disease reference group in many endpoints. All ACO groups present lower post-bronchodilator FEV1%pred, higher smoking pack-years history, and a different pattern of comorbidities. The prevalence of comorbidities is significantly and consistently higher in all ACO groups. Patients in the GINA/GOLD/30% group had the highest number of comorbid diseases, occurring more frequently than those in the non-ACO group (nine comorbidities), followed by the COPD + asthma < 40 group and the clinician’s diagnosis group (seven comorbidities), the Gibson’s group (five comorbidities), and the Spanish criteria group (two comorbidities). Noteworthy is the higher rate of hypertension, ischemic heart disease, myocardial infarction, osteoporosis, GERD, and chronic sinusitis. All the diseases mentioned co-occur in most ACO groups. The higher prevalence of cardiovascular diseases, osteoporosis, and GERD in ACO patients compared to those with asthma or COPD alone is a well-known observation and was confirmed in several epidemiological studies [38,39,40]. A new observation is the consistently higher prevalence of chronic sinusitis in ACO groups based on definitions that more accurately describe asthma attributes, i.e., GINA/GOLD/30%, COPD + asthma < 40, and Spanish criteria groups. The mechanism of the relationship between chronic sinusitis and ACO is not fully understood. Epidemiological studies have reported a significant association between allergic rhinitis and ACO and a higher prevalence of allergic rhinitis in ACO compared to COPD alone [39]. Most likely, it is the causal relationship between rhinosinusitis and asthma that is key to the co-occurrence of rhinosinusitis and ACO. The etiopathologic relationship between chronic sinusitis, both allergic and non-allergic, and asthma is well established [26,27,28]. We believe that the associations presented may shed light on the observed relationship between chronic sinusitis and ACO.

In the final analysis, we compared the prevalence of comorbidities between all patients with ACO and those without ACO (all-ACO vs. non-ACO). This approach is also innovative, as it compares ACO patients to a reference group of patients with obstructive diseases rather than separately to asthma or COPD. The results are consistent in large part with studies comparing ACO with asthma or COPD [38,39,40] and confirm a higher prevalence of comorbidities in the ACO group. The higher burden of comorbidities observed among patients with ACO may reflect the complex pathophysiology and greater overall disease burden associated with the coexistence of asthma and COPD features. Several mechanisms may contribute to this observation. Persistent low-grade systemic inflammation, characteristic of chronic airway diseases, may promote the development or progression of extrapulmonary manifestations and contribute to a higher prevalence of comorbid conditions. In addition, the overlap of asthma- and COPD-related inflammatory pathways may result in a broader inflammatory profile compared with either disease alone. Furthermore, patients with ACO may represent a subgroup with greater cumulative exposure to risk factors, including smoking, more severe and persistent airflow limitation, and longer disease duration, all of which may be associated with increased comorbidity burden. Long-term corticosteroid exposure, particularly systemic corticosteroid use or repeated courses of oral corticosteroids, may also contribute to the occurrence of selected comorbidities. Finally, accelerated biological aging, which has been suggested in chronic inflammatory respiratory diseases, may represent another potential mechanism linking ACO with a higher prevalence of age-related comorbid conditions. However, given the cross-sectional design of our study, these mechanisms should be considered as potential explanations rather than causal relationships. Further longitudinal studies are needed to clarify whether ACO independently contributes to the development of comorbidities or whether the increased comorbidity burden reflects the accumulation of shared risk factors and greater disease severity. The most common comorbidities with a higher prevalence in the ACO group are cardiovascular diseases, GERD, musculoskeletal disorders, osteoporosis, and insomnia. The prevalence of diabetes was also higher in the group of patients with ACO but was not statistically different from the group of patients without ACO. A distinctive observation in our study is the lower prevalence of heart failure in the ACO group, which is inconsistent with some other studies [39,40].

The study has some limitations. The most relevant appears to be the lack of a universally accepted definition of ACO. Different criteria for different ACO definitions may result in the emergence of clinically diverse patient populations. The definition of ACO, proposed by Gibson and Simpson in 2009 [13], emphasized the persistent airflow limitation. The only asthmatic quality included in this definition was the increased variability of airflow, associated with bronchial hyperresponsiveness (BHR). Nonetheless, BHR is also a common feature in COPD. As a result, the Gibson–Simpson definition may describe not only ACO subjects but “pure” COPD patients as well. All other definitions also contain the principal COPD criterion, i.e., persistent obstruction, but concurrently incorporate more characteristics of asthma. The most balanced one seems to be the GINA/GOLD definition [3]. It covers eleven features of COPD and eleven features of asthma so that it better captures the asthmatic face of the overlap. The Spanish definition is close in this respect. It not only emphasizes BHR but also considers sputum eosinophilia and atopy or early diagnosis of asthma [10]. To some extent, a similar concept is behind the COPD + asthma < 40 definition, where, in addition to the diagnosis of COPD, a prior diagnosis of asthma is required [7,11,12]. The dissimilarities between differently defined ACO groups limit the ability to accurately characterize the ACO phenomenon. Multiple ACO definitions and variations in clinical practice may lead to patient classification bias that may impact study endpoints. This applies specifically to the physician’s diagnosis group, where the diagnosis is made based solely on the doctor’s experience and is not supported by clearly defined ACO diagnostic criteria. The variations in clinical practice across participating centers may impact not only ACO phenotyping but also other clinical outcomes, specifically comorbidity screening and treatment decisions.

An additional limitation of the study is the large number of statistical comparisons performed. No formal adjustment for multiple testing was applied because the analysis was exploratory in nature. Consequently, some statistically significant associations may represent type I error and should be interpreted cautiously until confirmed in independent cohorts.

## 5. Summary

Differences in the definitions of ACO result in different populations. ACO groups differ in terms of comorbidities, lung function, smoking history, and age. Because all ACO definitions include the mandatory condition of irreversible obstruction, the quality that effectively distinguishes ACO groups is the inclusion of different asthma criteria. Chronic sinusitis is a comorbidity that consistently occurs more frequently in ACO groups defined by more precise criteria of asthma, i.e., GINA/GOLD/30% group, Spanish criteria group, and COPD + asthma < 40 yrs group. This may be related to the well-established association between rhinosinusitis and asthma.

The ACO groups differ from the other obstructive disease group by having a higher number of pack-years, worse lung function, and more comorbidities. Comorbidities were consistently more numerous in the individual ACO groups compared to the other obstructive disease group.

A similar observation regarding comorbidities applies to the all-ACO group. The most common comorbidities in the combined group of all-ACO patients were hypertension, coronary heart disease, GERD, musculoskeletal disorders, osteoporosis, diabetes, and chronic sinusitis. We also noted a previously unreported increased prevalence of genitourinary and ophthalmic diseases in patients with ACO.

## 6. Conclusions

The definitions of ACO used in clinical practice vary widely and describe different populations. ACO populations vary in terms of comorbidities, lung function, smoking history, and age.

In this exploratory analysis, chronic sinusitis was more frequently observed in ACO groups defined by more precise asthma criteria.

Patients with ACO differ in their clinical outcomes from patients with other obstructive pulmonary diseases. The most common comorbidities in patients with ACO are hypertension, coronary heart disease, GERD, musculoskeletal disorders, osteoporosis, diabetes, and chronic sinusitis.

## Figures and Tables

**Table 1:** The key diagnostic criteria comprising the various ACO definitions based on the classification used in the Barczyk et al. study [9].

Label Assigned to ACO Definition	The Key Diagnostic Criteria of ACO	Comments
GINA/GOLD/30%	ACO is considered when a patient presents with three or more features suggestive of asthma and three or more features suggestive of COPD among the 11 clinical characteristics used to differentiate between these conditions. Importantly, the number of features supporting asthma and COPD should be approximately similar [3].	As no explanation for the term “similar” was provided in the original document, we used our own consensus definition, based on the calculation of the relative difference between the number of specific features. The threshold of 30% was arbitrarily chosen for discriminating ACO from asthma or COPD; i.e., patients were qualified as ACO if their relative difference in the number of asthma and COPD features was lower than 30%.
The Spanish criteria	The criteria include 6 diagnostic criteria (3 major, 3 minor), with a combination of 2 major or 1 major and 2 minor criteria required for ACO diagnosis [10].	As the Spanish criteria refer only to the population with COPD and not chronic airway disease in general, we additionally used history of smoking and post-bronchodilator FEV1/FVC < 70% as mandatory for COPD diagnosis.
COPD + asthma < 40 yrs	ACO defined as current COPD diagnosis in a patient with asthma recognized before the age of 40 years [7,11,12].	As these criteria can be used only for patients with COPD, an additional criterion for COPD diagnosis (positive smoking history and postbronchodilator FEV1/FVC < 70%) was added for the purpose of the current study, as previously applied for the Spanish criteria.
Gibson’s criteria	ACO defined as symptoms of increased variability of airflow and incompletely reversible airflow obstruction was recognized when a patient had a history of smoking, postbronchodilator FEV1%FVC < 70%, and a history of at least one positive result of bronchial reversibility test [13].	
Physician’s diagnosis	ACO diagnosis based on clinical experience and judgment, i.e., when, in the opinion of the attending respiratory specialist, the proper diagnosis in his/her patient was asthma and COPD.	

**Table 2:** Comorbidities included in the final dataset.

1	coronary artery disease
2	myocardial infarction
3	hypertension
4	heart failure
5	cor pulmonale (pulmonary heart disease) or pulmonary hypertension
6	other cardiovascular diseases
7	pulmonary embolism
8	tuberculosis
9	bronchiectasis
10	sarcoidosis
11	eosinophilic granulomatosis with polyangiitis—EGPA (formerly named Churg–Strauss syndrome)
12	allergic bronchopulmonary aspergillosis (ABPA)
13	interstitial lung diseases
14	obstructive sleep apnea
15	other lung disease
16	gastroesophageal reflux disease (GERD)
17	other than GERD gastrointestinal (GI) disease
18	genitourinary diseases
19	neurological diseases
20	musculoskeletal disorders
21	osteoporosis
22	anemia
23	polycythemia
24	other hematologic diseases
25	diabetes
26	autoimmune diseases
27	chronic sinusitis
28	other otolaryngologic diseases
29	ophthalmic diseases
30	oncologic diseases
31	endocrine diseases
32	depression
33	insomnia

**Table 3:** The data on age, severity of airflow limitation, and smoking history in the ACO groups and the non-ACO groups.

	Age	*p*-Value ^†^	Post-BD FEV1%pred	*p*-Value ^†^	PY	*p*-Value ^†^
non-ACO	61.9 (12.1)		68.6 (24.6)		16.9 (22.7)	
GINA/GOLD/30%	63.5 (9.7)	0.23	64.1 (19.0)	0.033	19.3 (22.8)	0.014
Spanish criteria	57.8 (10.8)	0.006	60.5 (17.6)	0.009	22.4 (19.3)	<0.001
COPD + asthma < 40 yrs	60.4 (11.8)	0.12	56.9 (17.3)	<0.001	23.3 (18.3)	<0.001
Clinician’s diagnosis	64.8 (9.9)	0.001	58.5 (19.1)	<0.001	20.2 (21.9)	<0.001
Gibson’s criteria	64.3 (9.7)	0.036	53.8 (16.3)	<0.001	30.7 (22.1)	<0.001

Data are presented as mean (SD). PY = pack-years. A *p*-value of less than 0.05 was taken as the threshold of statistical significance. ^†^ vs. non-ACO.

## Data Availability

Data available from researchers.

## Supplementary Materials

The following supporting information can be downloaded at https://www.mdpi.com/article/10.3390/arm94050062/s1: Table S1: The prevalence of all comorbidities included in the dataset in the ACO groups, corresponding other-ACO groups and the non-ACO group. Disease prevalence is expressed as a percentage of patients in a group diagnosed with a particular disease [%]. Table S2: The prevalence of all comorbidities included in the dataset in the all-ACO group and the non-ACO group. Disease prevalence is expressed as a percentage of patients in a group diagnosed with a particular disease [%].
