# Supplementary material for: Patterns of Comorbidity in Asthma–COPD Overlap Cohort According to Various ACO Definitions
Source: Adv Respir Med. 2026 Aug 31;94(5):62. doi: 10.3390/arm94050062 (PMC13600012; doi:10.3390/arm94050062)
Supplement: Supplementary file 1 [file arm-94-00062-s001.zip › arm-4434624-supplementary.pdf]

advances in respiratory medicine

MDPI

Supplementary Materials

Table S1. The prevalence of all comorbidities included in the dataset in the ACO groups, corresponding other-ACO groups and the non-ACO group. Disease prevalence is expressed as a percentage of patients in a group diagnosed with a particular disease [%].

	Clinician's diagnosis [%]	*corresponding other-ACO [%]*	*COPD+asthma<40 [%]*	*corresponding other-ACO [%]*	*Spanish criteria [%]*	*corresponding other-ACO [%]*	*Gibson's criteria [%]*	*corresponding other-ACO [%]*	*GINA/GOLD 30% [%]*	*corresponding other-ACO [%]*	*non-ACO [%]*
coronary artery disease	*30.4[(b)]*	*24.4*	*24.2*	*28.7*	*19.7*	*28.7*	*23,6*	*30.0*	*26.7*	*28.1*	*20.6*
myocardial infarction	*7.8*	*7.9*	*11.3[(b)]*	*6.8*	*9.8*	*7.6*	*9.2[(b)]*	*7.0*	*4.1[(a)]*	*9.6*	*5.3*
hypertension	*61.2[(b)]*	*62.8*	*61.3[(b)]*	*62.1*	*57.4*	*62.5*	*61.5[(b)]*	*62.1*	*65.7[(b)]*	*60.1*	*50.1*
heart failure	*10.1*	*7.9*	*9.7*	*8.9*	*3.3[(b)]*	*9.9*	*7.7[(b)]*	*9.9*	*9.3*	*9.0*	*12.8*
cor pulmonale or pulmonary hypertension	*3.4*	*0.8*	*2.4*	*2.2*	*0*	*2.5*	*1.0*	*2.9*	*0.1*	*3.0*	*3.1*
other cardiovascular diseases	*7.8*	*11.6*	*8.9*	*9.7*	*4.9*	*10.1*	*10.8*	*8.8*	*12.2*	*8.2*	*9.6*
pulmonary embolism	*1.4*	*0*	*0*	*1.0*	*0*	*0.8*	*0*	*1.2*	*1.2*	*0.6*	*0.8*
tuberculosis	*4.1*	*4.1*	*2.4*	*4.6*	*1.6*	*4.4*	*6.7[(a, b)]*	*2.6*	*1.2[(a)]*	*5.5*	*2.6*
bronchiectasis	*3.0*	*2.1*	*4.0*	*2.2*	*3.3*	*2.5*	*2.1*	*2.9*	*1.7*	*3.0*	*2.9*
sarcoidosis	*0*	*0.8*	*0*	*0.5*	*0*	*0.4*	*0.5*	*0.3*	*0.6*	*0.3*	*1.1*
eosinophilic granulomatosis with polyangiitis - EGPA	*0*	*0.4*	*0*	*0.2*	*0*	*0.2*	*0*	*0.3*	*0.6*	*0*	*0.1*
allergic bronchopulmonary aspergillosis - ABPA	*0.3*	*0*	*0*	*0.2*	*0*	*0.2*	*0*	*0.3*	*0.6*	*0*	*0.1*
other interstitial lung diseases	*0.3*	*0.4*	*0*	*0.5*	*0*	*0.4*	*0*	*0.6*	*0.6*	*0.3*	*0.9*
obstructive sleep apnea	*3.4*	*3.7*	*3.2*	*3.6*	*1.6*	*3.8*	*3.6*	*3.5*	*4.1*	*3.3*	*2.5*
other lung disease	*2.0*	*1.7*	*1.6*	*1.9*	*0*	*2.1*	*2.1*	*1.8*	*2.3*	*1.6*	*3.3*
gastroesophageal reflux disease - GERD	*22.3[(b)]*	*23.1*	*25.0[(b)]*	*22.0*	*34.4[(a, b)]*	*21.2*	*17.4[(a)]*	*25.7*	*27.9[(b)]*	*20.2*	*16.4*
other than GERD GI diseases	*9.5*	*10.7*	*7.3*	*10.9*	*6.6*	*10.5*	*10.3*	*9.9*	*14.5[(a, b)]*	*7.9*	*7.9*
genitourinary diseases	*8.5*	*12.8*	*8.1*	*11.1*	*3.3*	*11.3*	*11.8[(b)]*	*9.6*	*12.8[(b)]*	*9.3*	*6.1*
neurological diseases	*4.7*	*5.8*	*4.8*	*5.3*	*1.6*	*5.7*	*4.1*	*5.8*	*6.4*	*4.6*	*5.0*
musculoskeletal disorders	*16.9[(b)]*	*13.2*	*12.9*	*15.9*	*9.8*	*15.9*	*14.9[(b)]*	*15.5*	*18.6[(b)]*	*13.7*	*9.6*
osteoporosis	*10.8[(b)]*	*8.3*	*12.1[(b)]*	*8.9*	*8.2*	*9.9*	*6.7*	*11.4*	*11.1[(b)]*	*9.0*	*5.0*
anemia	*0.7*	*2.1*	*0.8*	*1.5*	*0*	*1.5*	*0.5*	*1.8*	*2.9[(a)]*	*0.6*	*1.4*
polycythemia	*3.0[(a, b)]*	*0*	*2.4*	*1.5*	*3.3*	*1.5*	*0.5*	*2.3*	*1.2*	*1.9*	*0.8*
other hematologic diseases	*0.7*	*0.8*	*0.8*	*0.7*	*0*	*0.8*	*1.0*	*0.6*	*0.6*	*0.8*	*0.9*
diabetes	*15.9*	*16.9*	*14.5*	*16.9*	*14.8*	*16.6*	*17.4*	*15.7*	*17.4*	*15.9*	*13.5*
autoimmune diseases	*1.0*	*0.4*	*0.8*	*0.7*	*1.6*	*0.6*	*0.5*	*0.9*	*0*	*1.1*	*0.7*

chronic sinusitis	*10.8*	*11.6*	*17.7[a,b]*	*9.2*	*19.7[a,b]*	*10.1*	*10.3*	*11.7*	*16.9[a,b]*	*8.5*	*8.3*
other otolaryngologic diseases	*5.7*	*5.4*	*6.5*	*5.3*	*11.5*	*4.8*	*7.2*	*4.7*	*7.6*	*4.6*	*6.6*
ophthalmic diseases	*5.4*	*6.6*	*8.1[b]*	*5.3*	*6.6*	*5.9*	*5.1*	*6.4*	*7.6[b]*	*5.2*	*3.2*
oncology diseases	*3.7*	*5.0*	*5.7*	*3.9*	*4.9*	*4.2*	*4.1*	*4.4*	*4.7*	*4.1*	*4.2*
endocrine diseases	*8.1*	*9.5*	*8.1*	*8.9*	*9.8*	*8.6*	*9.7*	*8.2*	*13.4[a]*	*6.6*	*9.1*
depression	*6.8*	*5.4*	*8.9[b]*	*5.3*	*6.6*	*6.1*	*3.6*	*7.6*	*7.6*	*5.5*	*4.2*
insomnia	*7.8[b]*	*7.4*	*7.3*	*7.7*	*4.9*	*8.0*	*6.7*	*8.2*	*9.9[b]*	*6.6*	*3.6*

P value of less than 0.05 was taken as the threshold of statistical significance. Note: [a]difference between ACO group and other-ACO group statistically significant, [b]difference between ACO group and non-ACO group statistically significant.

Table S2. The prevalence of all comorbidities included in the dataset in the all-ACO group and the non-ACO group. Disease prevalence is expressed as a percentage of patients in a group diagnosed with a particular disease [%].

	all-ACO [%]	non-ACO [%]	P value
coronary artery disease	27.7	20.6	0.002
myocardial infarction	7.8	5.3	NS
hypertension	61.9	50.1	<0.001
heart failure	9.1	12.8	0.032
cor pulmonale or pulmonary hypertension	2.2	3.1	NS
other cardiovascular diseases	9.5	9.6	NS
pulmonary embolism	0.7	0.7	NS
tuberculosis	4.1	2.6	NS
bronchiectasis	2.6	2.9	NS
sarcoidosis	0.4	1.1	NS
eosinophilic granulomatosis with polyangiitis - EGPA	0.2	0.1	NS
allergic bronchopulmonary aspergillosis - ABPA	0.2	0.1	NS
other interstitial lung diseases	0.4	0.9	NS
obstructive sleep apnea	3.5	2.5	NS
other lung disease	1.9	3.3	NS
gastroesophageal reflux diseases - GERD	22.7	16.4	0.003
other than GERD GI diseases	10.0	7.9	NS
genitourinary diseases	10.4	6.1	0.003
neurological diseases	5.2	5.0	NS
musculoskeletal disorders	15.2	9.6	0.001
osteoporosis	9.7	4.9	<0.001
anemia	1.3	1.4	NS
polycythemia	1.7	0.7	NS
other hematologic diseases	0.7	0.9	NS
diabetes	16.4	13.4	NS
autoimmune diseases	0.7	0.7	NS
chronic sinusitis	11.2	8.3	NS
other otolaryngologic diseases	5.6	6.6	NS
ophthalmic diseases	5.9	3.2	0.011
oncology diseases	4.3	4.2	NS
endocrine diseases	8.7	9.1	NS

depression	6.1	4.2	NS
insomnia	7.6	3.6	<0.001

NS = statistically non-significant. P value of less than 0.05 was taken as the threshold of statistical significance.

Disclaimer/Publisher's Note: The statements, opinions and data contained in all publications are solely those of the individual author(s) and contributor(s) and not of MDPI and/or the editor(s). MDPI and/or the editor(s) disclaim responsibility for any injury to people or property resulting from any ideas, methods, instructions or products referred to in the content.

https://doi.org/10.3390/xxxxx
